# Unique Periodic Rings Composed of Fractal-Growth Dendritic Branching in Poly(p-dioxanone)

**DOI:** 10.3390/polym14040805

**Published:** 2022-02-19

**Authors:** Kuan-Ying Huang, Eamor M. Woo, Selvaraj Nagarajan

**Affiliations:** Department of Chemical Engineering, National Cheng Kung University No. 1, University Road, Tainan 701-01, Taiwan; minnie850422@gmail.com (K.-Y.H.); nagarajan.tech@gmail.com (S.N.)

**Keywords:** poly(p-dioxanone), poly(p-vinyl phenol), fractal, banded dendrites, self-assembly

## Abstract

Amorphous poly(p-vinyl phenol) (PVPh) was added into semicrystalline poly(p-dioxanone) (PPDO) to induce a uniquely novel dendritic/ringed morphology. Polarized-light optical, atomic-force and scanning electron microscopy (POM, AFM, and SEM) techniques were used to observe the crystal arrangement of a uniquely peculiar cactus-like dendritic PPDO spherulite, with periodic ring bands not continuingly circular such as those conventional types reported in the literature, but discrete and detached to self-assemble on each of the branches of the lobs. Correlations and responsible mechanisms for the formation of this peculiar banded-dendritic structure were analyzed. The periodic bands on the top surface and interior of each of the cactus-like lobs were discussed. The banded pattern was composed of feather-like lamellae in random fractals alternately varying their orientations from the radial direction to the tangential one. The tail ends of lamellae at the growth front spawned nucleation cites for new branches; in cycles, the feather-like lamellae self-divided into multiple branches following the Fibonacci sequence to fill the ever-expanding space with the increase of the radius. The branching fractals in the sequence and the periodic ring-banded assembly on each of the segregated lobs of cactus-like dendrites were the key characteristics leading to the formation of this unique dendritic/ringed PPDO spherulite.

## 1. Introduction

By adjusting the ratio of the polymers and crystallization temperature, many polymer blends exhibit diverse crystallization morphologies, such as Maltese cross spherulites, ring-banded spherulites, dendritic spherulites, hexagonal spherulites [1], feather-like spherulites [2], seaweed spherulites [3], and the unique Janus-face spherulites [4,5]. However, in recent decades, the formation mechanism of periodic ring-banded spherulites was interpreted controversially. Recently, based on novel research approaches, the innovative insights reveal that the periodic ring-banded spherulites are composed of the lamellae changing the orientation alternatively instead of continuous helical twisting lamellae [6].

Poly(p-dioxanone) (PPDO) is a synthetic polyester used in numerous biomedical applications, such as surgical sutures, cardiovascular applications, and drug carriers, due to its distinguished biodegradability and biocompatibility [7]. Most earlier researches of PPDO focused on changes in the physical and mechanical properties during a hydrolysis process [8]. However, these properties of semicrystalline polymers are greatly related to crystallization behavior and will affect the survival of cells in vivo in the human body [9,10,11]. Crystallization of PPDO usually leads to periodically ring-banded spherulites, which sensitively change with the molecular weight and crystallization temperature [8,12]. Therefore, PPDO, which is easily hydrolyzed, needs to be synthesized in copolymers [9] or blended with other polymers to make up for its defects. Blending is usually the simpler way to improve or custom-adjust the physical and mechanical properties of polymers. There are many studies on the PPDO blend systems that investigate the miscibility, effect of different intermolecular interactions, and the crystalline behaviors, etc., with ample examples such as PPDO/poly(ʟ-lactic acid) (PLLA) [13], PPDO/poly(lactic-co-glycolic acid) (PLGA) [14], PPDO/poly(3-hydroxybutyrate) (PHB) [15], PPDO/poly(ethylene succinate) (PESu) [16], PESu/PVPh [17], and PPDO/PVPh [18,19], etc. PVPh is an amorphous polymer with a high glass transition temperature and is used in tissue engineering due to its biocompatibility [20]. PVPh, having a hydroxyl group at the para-position of the pendant phenyl ring, forms miscible blends with some polymers, such as poly(trimethylene terephthalate) (PTT) [21], poly(3-hydroxybutyrate) (PHB) [22,23], poly(ethylene oxide) (PEO) [24], PLLA [25], and PPDO [18,19], etc. The miscibility-induced changes in the physical properties of polymer blends containing PVPh are usually driven by the hydrogen-bonding interactions between the hydroxyl group of PVPh and the carbonyl group of the polyesters, as addressed in previous work on the phase behavior and specific interactions of PPDO/PVPh blends [18], confirming miscibility via T_g_-composition dependence and intermolecular interactions using FTIR analysis. However, there were no detailed investigations dealing with PPDO morphologies as they are crystallized from PPDO/PVPh blends.

Many semicrystalline polymers, when blended with strongly interacting hydroxyl-containing PVPh, may exhibit dendritic spherulites packed with feather-like lamellae [26,27,28]. More peculiarly, ring bands can be superimposed on the fibrous dendrites to collectively form banded dendrites in final aggregated assemblies. Thus, clearly, banded stripes are not necessarily continuous in the entire circumferential rings of the spherulites; instead, the bands are divided into isolated stripes contained in discrete dendrites, which originate from a common nucleus center. The objective of this work was to probe the mechanisms and assembly routes that finally lead to such peculiar dendritic ring bands in crystal aggregations. PPDO diluted by miscible PVPh serves as an ideal model for such investigation. Furthermore, without incorporation of PVPh, neat PPDO’s periodically ring-banded spherulites are usually packed with compact and highly squeezed PPDO lamellae, which might be difficult to analyze. This study aimed to investigate the influence of PVPh content on PPDO assembly morphology. Furthermore, the crystalline arrangement leading to the unique dendritic-ringed spherulites was probed in detail. 

## 2. Experimental

### 2.1. Materials and Preparation

PPDO was purchased from Sigma-Aldrich, Inc. (St. Louis, MO, USA), with viscosity = 1.5–2.2 dL/g (measured by dissolving in hexafluoroisopropanol in 0.1% (*w*/*v*)), T_g_ = −9.5 °C, and T_m_ = 104.8 °C. According to AJ Muller et al.’s work [8], they suggested the parameters of K and a for the Mark-Houwink equation can be used as follows: [η] = KM^a^_v_, where a = 0.63 and K = 79 × 10^3^ cm^3^g^−1^. As only the data of viscosity = 1.5–2.2 dL/g is provided by the supplier, one can estimate the viscosity-average molecular weights of PPDO used in this work using the calculated results provided in an earlier work by Bai et al. [29]. Consistently, according to both literature papers, the estimated M_v_ of PPDO is between 5.6–9.0 × 10^4^ g/mol.

PVPh was purchased from Polysciences, Inc. (Warrington, PA, USA), with M_w_ = 22,000 g/mol and T_g_ = 150.5 °C. For sample preparation, PPDO and PVPh (of specific wt. ratios) were co-dissolved into p-dioxane to 4 wt.% polymer solution. The homogeneous solution was then cast on the glass slide as thin films and dried by placing at 30 °C in a vacuum oven to remove the residual solvent. For thermal treatments, film samples were first heated to maximum melting temperature (T_max_) for 2 min on top of a hotplate to erase the thermal history, and then removed rapidly from the hotplate to a temperature-controlled hot stage (temperature precision ± 0.5 °C) being preset, respectively, at different crystallization temperatures for full crystallization. 

A novel objective of introducing PVPh into PPDO during crystallization was that the PVPh component could be easily removed to better expose the inner lamellae of the PPDO hierarchical structures. From the crystallized PPDO/PVPh mixtures, selective etching could be performed to remove the amorphous PVPh, leaving a much better-enhanced morphology contrast in the crystallized PPDO skeletons for convenient analysis of the assembly mechanism. Furthermore, to analyze the interior lamellar assembly in crystallized specimens, fracturing across film thickness was performed by rapidly quenching into a liquid nitrogen bath prior to stress-breaking the films. In order to obtain clearer crystal assembly patterns on the top surface and interiors of PPDO spherulites, methanol was used for selectively etching the amorphous PVPh constituent off before the samples were analyzed using AFM and SEM characterization. PPDO is much less soluble in methanol than PVPh; therefore, methanol was chosen as an ideal selective etching agent for removing the amorphous PVPh constituent from the crystallized PPDO/PVPh specimens.

### 2.2. Apparatus

Differential scanning calorimeter (DSC). A differential scanning calorimeter (Perkin-Elmer, Diamond DSC) equipped with a mechanical intra-cooler for quenching was used. Samples were uniformized in DSC cells by heating to 200 °C, held for 2 min, and rapidly quenching to −40 °C, then scanned from −40 °C to above T_m_ at a heating rate of 20 °C min^−1^ in order to observe T_g_. A continuous nitrogen flow in the DSC sample cell was maintained.

Polarized-light optical microscopy (POM, Nikon Optiphot-2), with a Nikon Digital Sight (DS-U1) camera and a microscopic hot stage (Linkam THMS-600 with T95 temperature programmer), was used. A wavelength-tint plate (530 nm) was inserted in POM to make contrast interference colors for all POM graphs.

Fourier-transform infrared spectroscopy (FTIR). Fourier-transform infrared (Nicolet 6700) was used for investigating molecular interactions between the constituents. Spectra were obtained at <1 cm^−1^ resolution, and averages of spectra were obtained from 32 scans in the standard wavenumber range of 400 to 4000 cm^−1^. Blend samples for IR measurements were cast as thin films with uniform thickness directly on KBr pellets at room temperature.

Atomic-force microscopy (AFM). The intermittent tapping mode of AFM (diCaliber, Veeco Co., Santa Barbara, CA, USA) with a silicon-tip (f = 70 kHz, r = 10 nm) was used to observe the topology of spherulites and the measurement of height profiles. The largest scan range of the scanner was 150 × 150 μm, and the smallest scan range could be down to 5 × 5 μm for larger magnifications on selected areas of interest.

High-resolution field-emission scanning electron microscopy (Hitachi SU8010, HR-FESEM) was used. Samples were coated with platinum using vacuum sputtering (10 mA, 300 s) prior to SEM observation.

## 3. Results and Discussion

**Figure 1:**
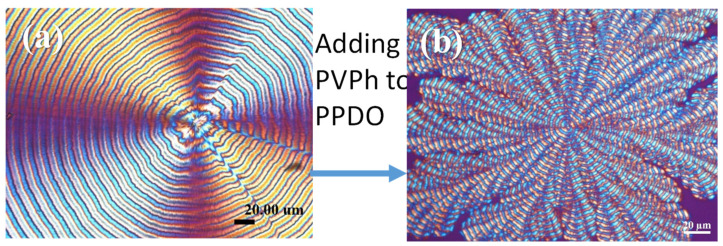
POM graphs of (**a**) neat PPDO vs. (**b**) PPDO in PPDO/PVPh blend (75/25), both at T_c_ = 78 °C for a period of 3–4 days till full crystallization.

Figure 1 show the POM of neat PPDO vs. PPDO crystallized from PPDO/PVPh blend (75/25), both at T_c_ = 78 °C. By adding a sufficient amount of PVPh in PPDO, the crystallized PPDO from the blend exhibited a systematic change in morphology from well-rounded circular (or continuous spirals) into octopus-arm-like dendrites where each of the “octopus-arm dendrites” displayed discrete ring bands along the lengthwise direction. To discriminate these from the regular ring-banded spherulites, this unique type were labeled as « dendritic/ringed » spherulites. With the increase of PVPh content in the PPDO/PVPh mixture, the original circularly ring-banded pattern (Figure 1a) gradually transformed to a highly dendritic one with multiple arms (or lobs) radiating out from a common center as shown in Figure 1b. Peculiarly, the ring bands persisted in each of the dendritic arms, with clearly interfacial boundaries between successive arms. At PVPh content = 10 wt.%, the PPDO dendrites were fine and crowded, with the inter-arm boundaries not clearly discernible. However, with the PVPh content reaching 20 wt.% or above, the dendrites became wider and interfacial boundaries became more obvious, resembling the petals of a sunflower. With PVPh content = 25% (Figure 1b), the petal-like pattern became quite clear, with each of the petals (arms) discrete and discernible from neighboring ones. Note that for PPDO/PVPh (80/20 or 75/25), it took a long time to develop spherulites at high T_c_ = 78 °C or 80 °C; therefore, samples for POM were usually kept at T_c_ for 3–4 days until full crystallization prior to morphology characterization. On each of the arms, ring bands were aligned independently with no connection to surrounding arms, meaning that the ring bands were no longer circular and continuous but discontinuous and confined within the arms. The specimens of PPDO/PVPh (75/25) were designated for in-depth analysis for assembly mechanisms leading to this unusual dendritic-banding pattern.

At either higher T_c_’s or greater contents of PVPh in PPDO/PVPh, the morphology exhibited a systematic change from original well-rounded bands into more dendritic forms with bands on the numerous individual dendrites. For brevity, the variation trend with respect to compositions is illustrated in the Supporting Info. Figures S1 and S2. Hydrogen-bonding interaction and phase miscibility were quite expected in the PPDO/PVPh mixtures, as shown in the DSC results of the Supporting Info. Figure S3. When the PVPh content (in PPOD/PVPh) reaches 20–30 wt.% or higher, the DSC traces show a single T_g_, which increases with the increase of PVPh (high-T_g_ component) content. The H-bonding between the –OH and –COO or –C-O- groups is proven in FTIR results in the Supporting Info. Figure S4. The carbonyl-stretching region and hydroxyl-stretching region are enlarged for convenience of inspection, which is in agreement with the literature for the same blend system [18]. The results show that interaction between PPDO and PVPh does exist, but there is still a higher intermolecular interaction between the PVPh molecules. From the present data, PPDO/PVPH can be safely confirmed as miscible in this molar-mass range. Normally, an increase in molecular weight may lead to a reduction of miscibility if their inter-molecular interactions are rather weak. For this pair of polymers, they have quite strong interactions via H-bonding, as proven in FTIR. Note, here in this work, we used a fixed Mw of PPDO to simplify the focused aims. The nature of ringed patterns of PPDO may vary with molecular weights. For poly(ʟ-lactic acid) (PLLA), a previous study [30] was conducted to evaluate the effects of molecular weight on banding patterns; indeed, as MW was varied, the banding patterns were different. 

**Figure 2:**
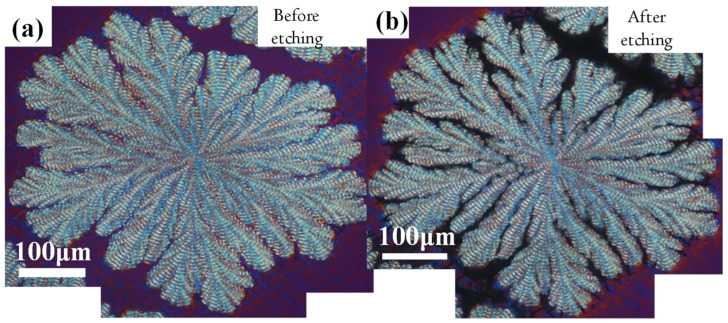
Dendritic-banded PPDO spherulites crystallized from PPDO/PVPh = 75/25, at T_c_ = 78 °C. Specimens (**a**) before etching and (**b**) after methanol-etching for 20 min.

Delicate etching techniques were adopted to remove PVPh from crystallized PPDO/PVPh films in order to enhance the morphology contrast for better analysis of the crystal packing of these peculiarly unique cactus-like banded-dendritic crystal aggregates. For in-depth morphology analysis, the well-defined dendritic rings in PPDO/PVPh (75/25) at T_c_ = 78 °C were chosen for thorough characterization using AFM and SEM. Yet, for clearer analysis of the dendritic-banded PPDO crystallized from PPDO/PVPh, the crystal-expelled amorphous PVPh had to be carefully etched off the crystalline PPDO spherulites. Methanol was found to be an ideal solvent, as it dissolved PVPh, leaving PPDO crystals almost intact. So, the crystallized film specimens were immersed in methanol with gentle rinsing/shaking for a certain time, dried off the solvent, and set ready for subsequent characterization. The POM micrographs for pristine (un-etched) and methanol-etched PPDO ringed dendrites crystallized from PPDO/PVPh (75/25) at 78 °C are shown in Figure 2 for specimens (a) before etching and (b) after etching, respectively. The objective of solvent-etching off the PVPh constituent was to undoubtedly prove that the palm-finger-like lobs in the banded dendrites are truly discrete and separate from each other. As shown, the dendrites before etching (Figure 2a) were more or less attached in borders; by contrast, the lobs and side branches in the cactus-like dendrites after methanol-solvent etching (Figure 2b) appear to be completely detached with clear crevices separating the lobs and side branches. 

**Figure 3:**
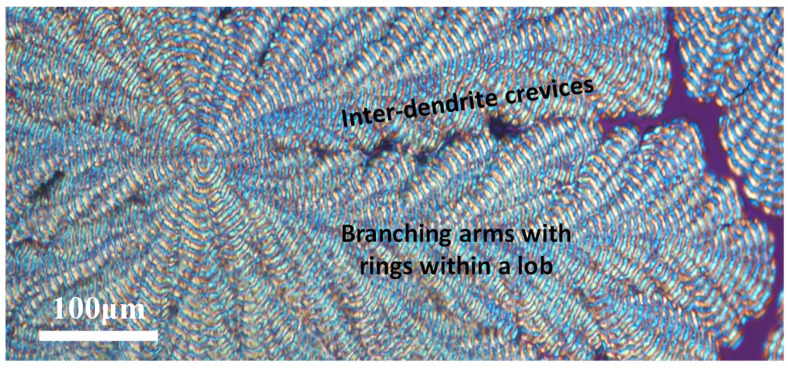
POM graph showing multiple-lob dendritic banded PPDO growing lengthwise (crystallized from PPDO/PVPh = 80/20, T_c_ = 80 °C.

Multiple POM images along a lengthwise direction were taken and then stacked together in order to reveal the entire long lob from the nucleus to the periphery of the PPDO banded-dendritic spherulite. Figure 3 show the POM graph displaying multiple-lob dendritic banded PPDO growing length-wise. In the zoom-in stacked POM graph, one can see clearly that there is a zig-zag boundary between successive lobs. Each lob is composed of fern-like side branches with periodic rings on each arm. In addition, each of the lobs is sub-divided into a main stalk from which numerous side branches grow with an oblique angle of ca. 45–60°, which varies with respect to the varying local crowdedness in growth. 

**Figure 4:**
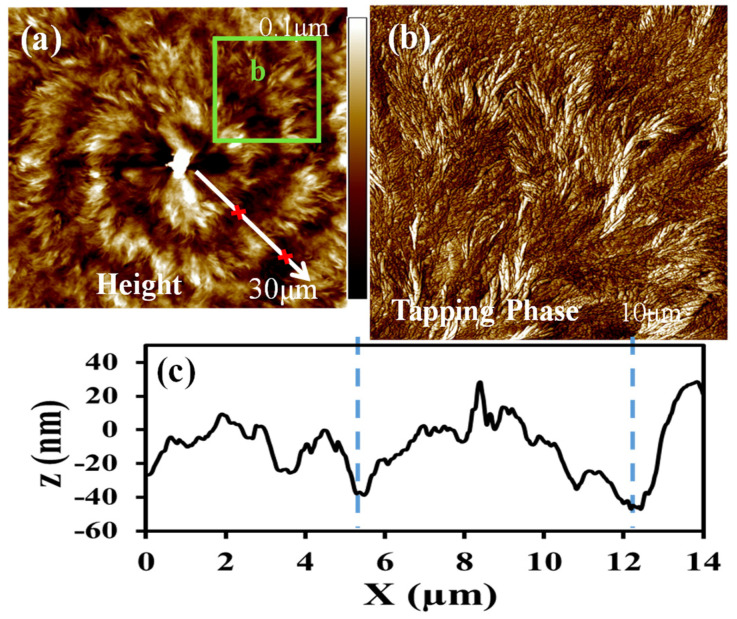
(**a**) AFM height images of the nucleus region of dendritic/ringed spherulites of PPDO/PVPh (75/25) blend crystallized at T_c_ = 78 °C, and (**b**) height profile along the radial direction [white arrow in Graph-(**a**): radial direction.], (**c**) height profile showing inter-band spacing = ca. 6 μm.

Dendrites with divisions of cactus-like lobs start to grow at locations farther away from the nucleus center of PPDO. Near the nucleus center, the ring bands still roughly remain circularly continuous. AFM analysis was conducted on regions near the nucleus in order to compare the differences of assembly between the regions near the nucleus and farther away from it, as shown in Figure 4. The first few (ca. 3) ring bands remain circularly continuous without dendritic patterns. Figure 4a,b show AFM images that reveal the topology of bands nearby the nucleus, with inter-band spacing = 7 μm, with height drop from ridge to valley = 70 nm, as shown in Figure 4c. The nucleus sheaf-crystals are obviously asymmetric, with the sheaf-crystals highly elongated in the length direction. The sheaf-crystals bend sharply at their tails into the circumferential direction for the first bands. It can be extrapolated that the second or third band is a repetitive cycle of the first band on the nucleus center. Square-b is zoomed-in (Figure 4b) for detailed analysis; it is obvious that the cycle of growth is composed of alternate radial lamellae extending for 3–4 μm, then abruptly bending/twisting in the tail end to the circumferential direction, in anti-clockwise sense (note here the CCD’s of AFM and SEM for recording the image are opposite—mirror images to each other). A new cycle then starts again with similar growth of the radial-oriented lamellae for 3–4 μm in the ridge, bending/twisting into the circumferential direction in the valley. Total inter-band spacing (one pitch – valley + ridge) is ca. 6.1 μm, as shown in Figure 4c. The growth cycles repeat in the same manner, with fractal branching to fill the ever-expanding space as the lamellae grow outward from the nucleus center. It can be shown later that the increase of the number of branches of the lobs of dendrites roughly follows the Fibonacci sequence, also reported earlier in many other polymer systems with dendritic growth [31,32,33,34,35].

**Figure 5:**
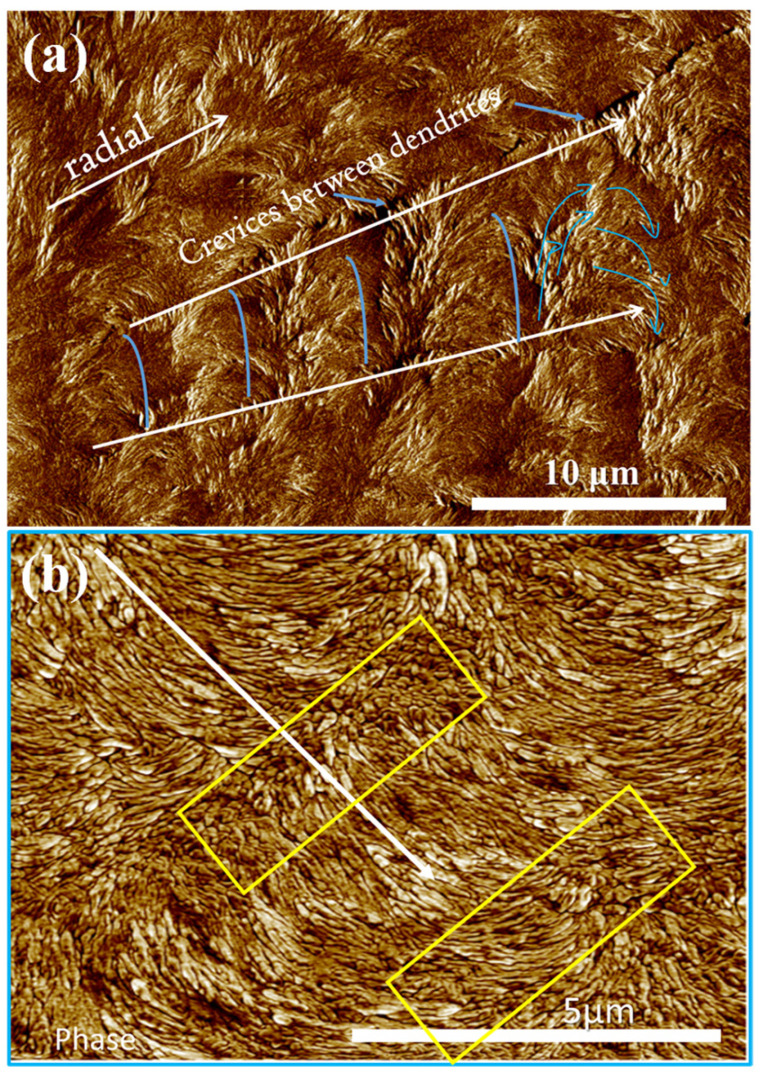
(**a**) Integrated AFM phase images, (**b**) integrated phase images for PPDO/PVPh = 75/25, at T_c_ = 78 °C, displaying individual rings along the lengthwise direction of the arms of the dendritic-rings, and interfacial crevices between dendrites.

Figure 5a,b show integrated AFM height and phase images for PPDO/PVPh = 75/25 crystallized at T_c_ = 78 °C (solvent-etched). AFM analysis provides better height profiles and image zoom-in than POM. The AFM results display that individual rings are formed along a lengthwise direction of the arm-finger branches of the banded dendritic PPDO. Furthermore, interfacial crevices between the neighboring lobs of dendrites are clearly seen in the AFM height images (Figure 5a). The zoomed-in phase image in Figure 5b more clearly reveal the differences between the alternate “valley” and “ridge” band in individual side branches of the finger-like banded-dendrites of PPDO. The yellow rectangles on the image mark the successive valley region; obviously, the discrete crystals (nano-meter sized) on the top surface of the valley bands are aligned in the circumferential direction. The “Ridge” band is outside the yellow rectangle marks, where all crystals are mostly aligned in the radial direction. The alignment change of crystals from radial to circumferential direction is abrupt and discontinuous and not a gradual twist such as that of a screw-helix. AFM analysis provides only crystal morphology views on the top surface. A three-dimensional interior dissection of these unique banded-dendrites will be expounded in the latter sections.

**Figure 6:**
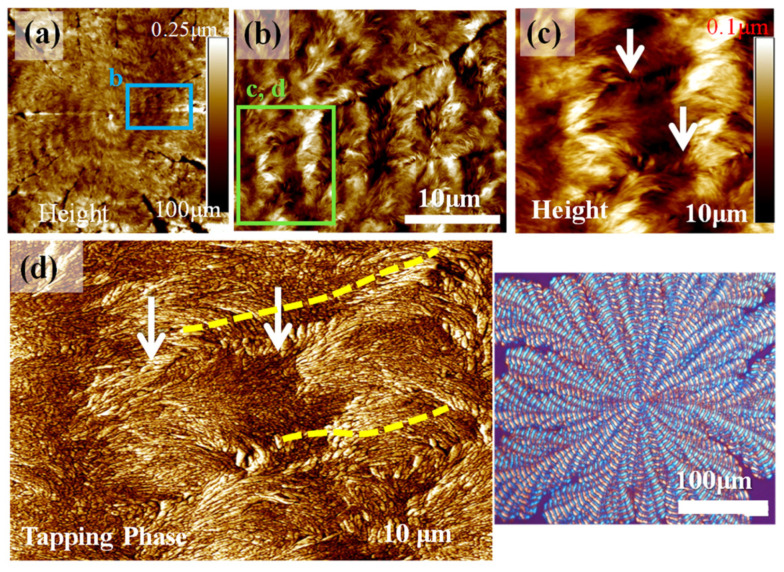
AFM height/phase images with stepwise zoom-in: (**a**) top surface of dendrite-ringed PPDO spherulite crystallized from PPDO/PVPh (75/25) at T_c_ = 78 °C, and (**b**) zoom-in revealing the microscopic branching pattern of the zoom-in to square-marked zone in graph (**a**), and (**c**) AFM height and (**d**) AFM phase images of bands in individual branches. (Inset: POM micrograph on lower right).

Obviously, bands are present independently in each of the side branches of lobs of the entire dendritic spherulites, and these bands are not smoothly circular around a common nucleus center. Instead, the bands adjust their orientations with respect to the direction of growth of each of the side branches. AFM analysis was further performed, and stepwise zoom-in morphology results are shown in Figure 6. Figure 6a is a low-mag. AFM height image showing the entire banded-dendritic spherulite. The blue-squared region is zoomed-in for analysis, as shown in Figure 6b. The green-squared box was further zoomed-in in Figure 6c as an AFM height image, where a valley (darker band) is sandwiched between two successive ridge bands (brighter bands). Obviously, the ridge is an elevated band in comparison to the lower valley, where both the ridge and valley have narrow crevices running between lamellar bundles that traverse from the ridge to valley bands. To inspect the lamellae on top surface, phase image zoom-in is shown in Figure 6d. In agreement with the analysis results in earlier Figure 5, the ridge band is populated with cilia fiber-like lamellae running in the radial direction, while the valley band is packed with discrete dot-like crystals running in the circumferential direction. From the radial-oriented ridge lamellae to the circumferential-oriented valley crystals, there is a narrow transition zone where the radial lamellae bend at a 75–90° angle and/or twist to merge into the circumferential crystal species.

**Figure 7:**
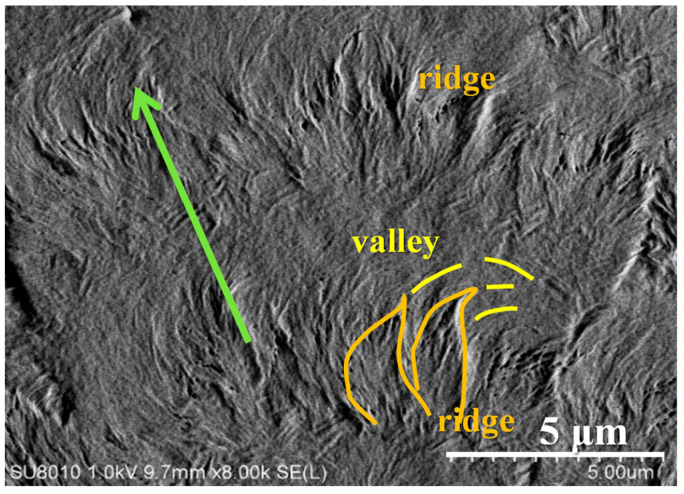
Zoom-in SEM micrograph of the top surface of PPDO/PVPh (75/25) blend crystallized at T_c_ = 78 °C. (Green arrows indicating the radial direction.).

By zooming into a specific spot of the side branch, one can see how lamellae are assembled on the alternate band period from ridge to valley. As supporting evidence to the above AFM, characterization using SEM was also performed. Figure 7 show that along a growth-axis direction, the lamellae are first along the growth direction to form a ridge region, then bend in the clockwise direction to the circumferential direction to form a valley stripe. This SEM result on the periodic bands in the side branches fully agrees with that of the AFM analysis, except that the alternate height variation is better discerned in the AFM analysis as discussed earlier. 

**Figure 8:**
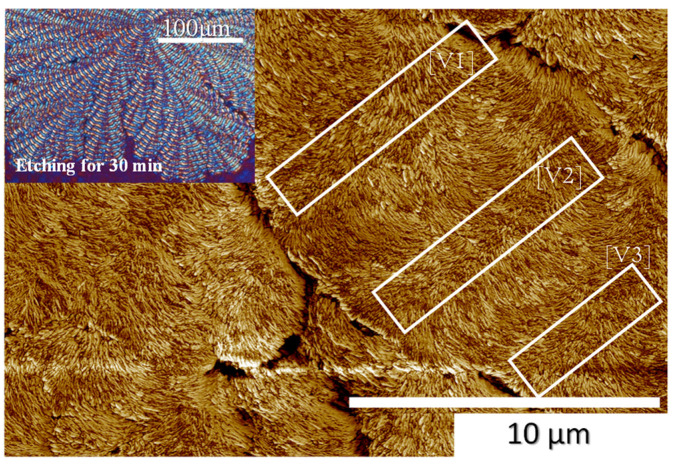
AFM zoom-in phase images (stacked to cover wider regions) for PPDO crystallized from PPDO/PVPh (80/20) at T_c_ = 80 °C. (POM inset on upper-left corner).

To further reinforce the proof of AFM analysis regarding crystal assembly leading to periodic bands, Figure 8 show AFM zoom-in phase images (with several images stacked to cover wider regions) for PPDO crystallized from PPDO/PVPh (80/20) at T_c_ = 80 °C. Inset on the upper left is a POM graph showing the entire banded-dendritic PPDO spherulite with multiple branches (or lobs) of the cactus-like dendrites. Once again, in agreement with the previous analysis, alternate crystal re-orientations are present in the cyclic growth leading to periodic bands. The ridge band is filled with cilia fber-like lamellae running in the radial direction; by contrast, the valley band is packed with discrete oval-shaped crystals running in the circumferential direction. A narrow transition zone exists between the ridge and valley, where the radial lamellae become much thinner and abruptly bend ca. a 75–90° angle and/or twist to merge into the circumferential crystal species. From the scale bar, the inter-band spacing (from mid-line of a ridge to mid-line of next ridge) is ca. 6–7 μm, with the dimension of the ridge region being 4 μm and that of the valley region being ca. 2 μm.

**Figure 9:**
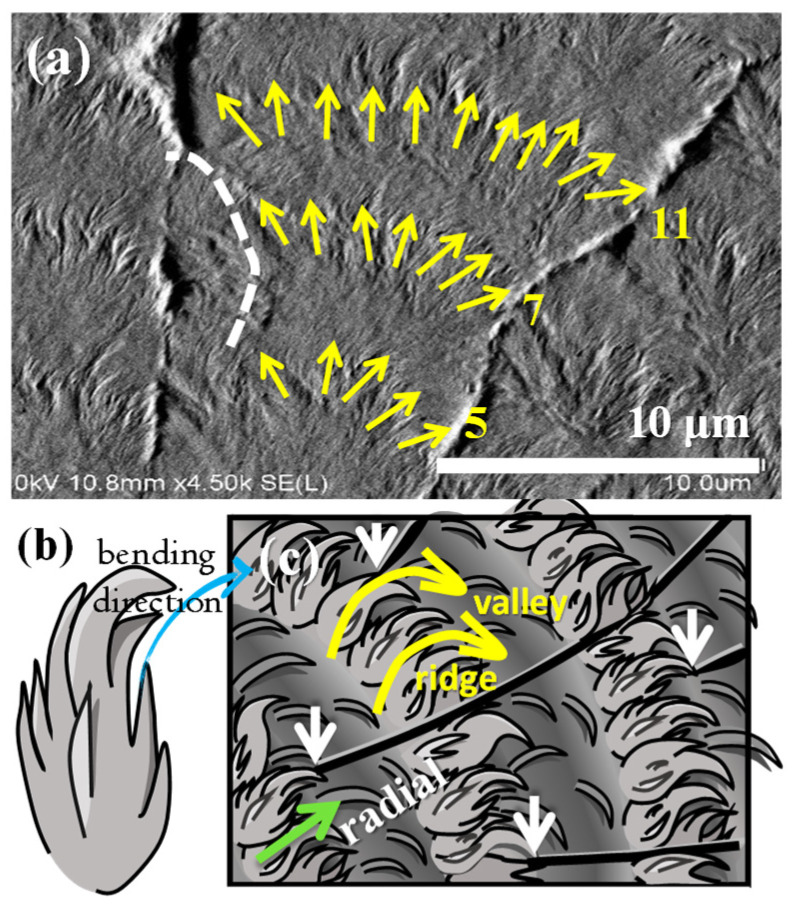
SEM micrograph of (**a**) top surface of banded-dendrites PPDO crystallized from PPDO/PVPh (75/25) blend at T_c_ = 78 °C, and (**b**) schematic for an individual fractal of crystal bundle in the ridge region, and (**c**) cycles of fractal-growth branches on the top surface of PPDO/PVPh (75/25).

Figure 9a show a zoomed-in SEM micrograph to cover the widthwise section of a lob, where yellow arrows mark the direction and number of branches as the lob grows outward. Fractal branching occurs periodically, and the branches splay out to occupy a fan-like sector. The number of fractals increases by the self-similar reproduction of branches in new cycles. Figure 9b show that the individual feather-like crystal bundle first grows in the radial direction for 3–4 μm; then, the available molten species drain out, becoming thinner and simultaneously bending/twisting abruptly in the clockwise sense into the circumferential direction. Figure 9c show that these fractals are placed in cycles to form an entire lob packed with alternate periodic bands, where the radial-oriented lamellae constitute the ridge, and the abrupt bend/twist lamellae, re-oriented in the circumferential direction, constitute the valley. Cycles repeat in the same growth manner. 

**Figure 10:**
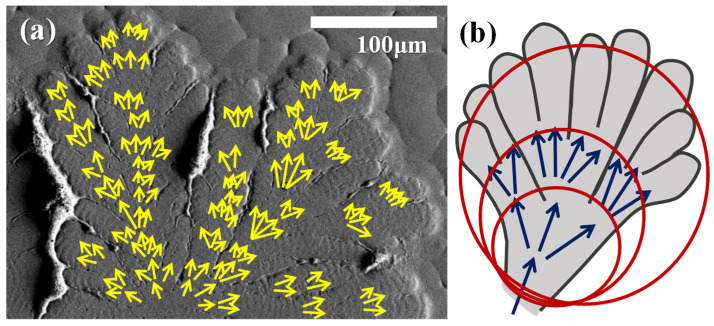
Dendritic-banded PPDO spherulite of PPDO/PVPh (75/25) blend crystallized at T_c_ = 78 °C, (**a**) SEM micrographs, and (**b**) schematic of fractal growth of dendrite-branching pattern (rings accompanying the intermittent fractals).

Branches grow away from the nucleus center and obviously increase with a certain pattern of sequence. The number of branches could be counted for quantification. The branches are traced in SEM graphs, as shown in Figure 10a. Near the nucleus center, the number of branches is limited due to space confinement; however, as the growth front propagates outward, the space increases with respect to the radius as R^2^ (2D) or R^3^ (3D). The growth front at each cycle then produces more sites for incubating branches, which multiply in number as R increases. The multiplication of branches in a certain sequence is illustrated in Figure 10b. The new branches are mostly or dominantly nucleated at the growth front of each of the growth cycles. Note that the cycle of growth-assembly cannot be depicted as a monotonous lamellar plate continuously twisting from edge-on to flat-on, as the growth of continuous twist lamellae without branching from the nucleus center cannot explain the crystal lamellae quickly filling the expanding space away from the nucleus. The experimental fact in this work is that there are numerous new branches spawned at the growth front of each of the new cycles. Such patterns of growth, accompanied by the Fibonacci-sequence expansion of branches, are also reported in many small-molecule compounds crystallized with periodic ring bands [36,37,38,39,40,41].

**Figure 11:**
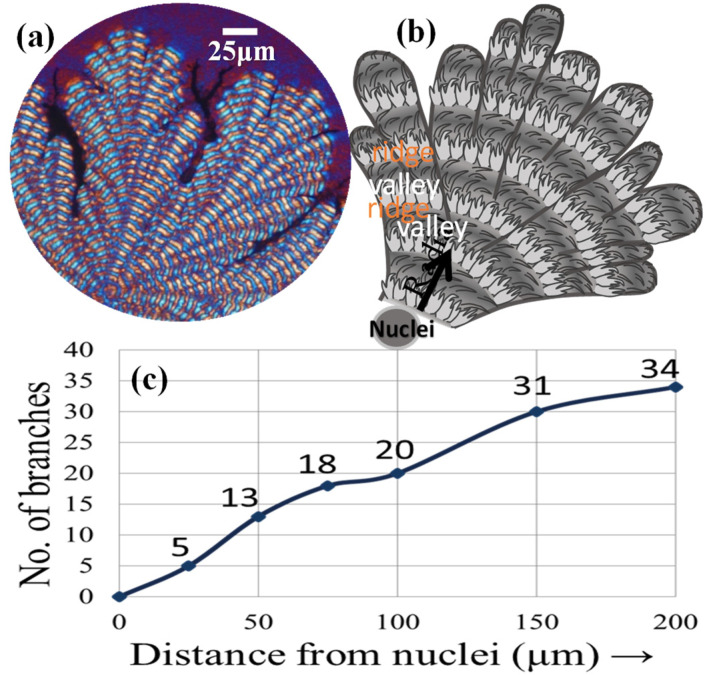
(**a**) POM graph for fractal-dendritic banded PPDO at T_c_ = 78 °C (from PPDO/PVPh (75/25), (**b**) schematic for packing of side branches, (**c**) number of branches increase with respect to distance from the nucleus center, approximately following the Fibonacci sequence.

Figure 11 show a chopped sector of the dendritic/ringed PPDO spherulite, containing three lobs of dendrites with 6–10 branches packed with ring bands in each lob. Ridges of each ring band are composed of fibrous branches in the radial direction, abruptly bending to the tangential direction upon reaching and merging into the valley. Both the POM graph (Figure 11a) and schematic (Figure 11b) summarize the key characteristics of the surface-relief morphology of dendritic-banded PPDO spherulite crystallized from the PPDO/PVPh (75/25) blend. An increase in the branch number as the growth starts from the nucleus center to the periphery is apparent, and this multiplication of branches is necessary to fill the ever-expanding space. The number of branches was estimated and plotted as a function of distance from the nucleus center, as shown in Figure 11c. The increasing number of branches of the lobs of dendrites roughly follows the Fibonacci sequence, which is also observed in many other polymer systems with dendritic growth [31,32,33,34,35].

**Figure 12:**
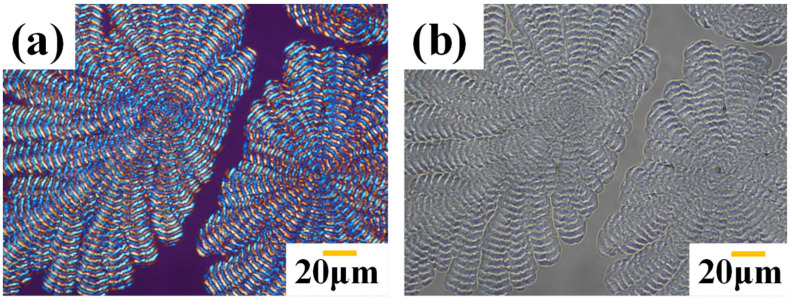
Comparative graphs of (**a**) POM and (**b**) OM for fractal-dendritic banded PPDO at T_c_ = 78 °C [crystallized from PPDO/PVPh (75/25)].

There is an issue concerning whether the crystallization of PPDO (from PPDO/PVPh blend) might result in the expulsion or segregation of the PVPh component from the PPDO lamellae or interphases in the aggregates. An earlier work dealing with a similarly miscible blend of poly(ε-caprolactone)/poly(phenyl methacrylate) (PCL/PPhMA) presented crystalline/amorphous phase separation during the crystallization process, and the amorphous phase of PPhMA was segregated with periodic intervals in the inter-lamellar regions of the ring-banded PCL [31,42]. This work is not directed to probe in detail how PVPh molecules diffuse/segregate within the morphology after the crystallization of PPDO; however, the issue can be preliminarily expounded from the POM results. POM/OM graphs of fractal-dendritic banded PPDO spherulites are given in Figure 12. Crystallization-driven phase separation is not evident in the PPDO/PVPh blend. Comparative graphs of the POM-(a) and OM-(b) show that the expulsion of amorphous PVPh does not occur at inter-spherulitic sites. In parallel with that, very sluggishly, slow crystallization (up to 4 days) of PPDO may segregate most PVPh molecules in the inter-lamellae region at the sub-micrometer level. As a result of strong H-bonding interactions and good miscibility (Figures S3 and S4), PPDO exhibits extremely slow crystallization growth, resulting in the amorphous PVPh not being detected in inter-spherulitic regions.

This work so far only focuses on the top-surface morphology of PPDO, which is in line with most previous investigations on PPDO in the literature. The POM birefringence patterns represent the statistic-averaged morphological arrangement of the entire sample volume and certainly not just the top surface. From several recent literature results on other polymers, such as poly(butylene adipate) (PBA), poly(ε-caprolactone) (PCL), and poly([3-hydroxybutyrate] (PHB), poly(nonamethylene terephthalate) (PNT), high-density polyethylene (PE), etc. [42,43,44,45,46,47,48,49], the periodic morphology is certainly not limited to top surfaces. Still, it also distributes in 3D bulks throughout the entire sample volume. Three-dimensional dissections on PPDO’s periodic assemblies are regarded to be essential in continuing future work.

## 4. Conclusions

In the PPDO/PVPh blend, the addition of amorphous yet strongly interacting PVPh into PPDO induces cactus-like dendrites packed with flower-petal-shape lobs, with periodic bands aligned on the individual side branches of each of the lobs. The periodic ring bands are thus not in smooth circular patterns as conventionally seen in most banded polymer spherulites but individually packed on each of branches of the cactus-like lobs with distinct detached boundaries. The unique morphology of banded dendrites only occurs when PPDO crystallizes in the presence of the PVPh diluent at content >20 wt.%, suggesting that the strongly interacting diluent has a dramatic effect on modulating the morphology of PPDO into this peculiar pattern. The first three to four periodic bands near the nucleus center are fully and continuingly circular without disrupted cusps; however, as the growth continues to spread away from the nucleus region, cactus-like lobs evolve and self-divide into dendritic branching structures with the ring bands being distributed and self-aligned on each of the lob-like side branches. Cycles of ring bands on each side branch are packed with alternate feather-like radial-oriented lamellae, alternately changing orientations in clockwise bending from the radial direction to the tangential direction and taper to dot-like crystals in the valley. Meanwhile, the lamellae at the growth front of the feather-like fractals randomly divide into multiple dendrites to fill the growing space with the increase of the radius of the spherulite. 

The PPDO crystalline morphology demonstrates that the branching of fractals and the periodically discontinuous crystalline assembly are the main characteristics leading to the formation of the dendritic ring-banded spherulites. The ring bands are present in each of the side branches of the lobs (assembled as the pattern of a fern leaf) of the dendritic PDDO spherulite. Depending on the growth direction of the lob and side branches, these numerous ring bands on each side branch also adjust their orientations, leading to a peculiar banded-dendritic PPDO aggregate. Each periodic ring band with alternate ridge and valley stripes is composed of cyclic growth of the radial-oriented lamellae, which bend/twist as they reach a length of ~ca. 4 μm and abruptly change the original radial direction to the circumferential (i.e., tangential) one. The cycles repeat in the same fractal pattern until the complete drainage of all available molten species upon reaching the periphery and impingement with the neighboring spherulites. Each of the lobs and side branches therein are detached from each other and individually oriented in slightly varying directions/orientations upon filling the space in the most compact way of nature’s random fractal pattern.

## Data Availability

Data are contained within the article and are available upon reasonable request.

## Supplementary Materials

The following supporting information can be downloaded at: https://www.mdpi.com/article/10.3390/polym14040805/s1, Figure S1. Effects of T_c_ and composition on POM birefringence patterns of PPDO crystallized from PPDO/PVPh blend; Figure S2. POM graphs of PPDO in PPDO/PVPh blend all crystallized at a same specific T_c_ = 78 °C of four different compositions: (a) 100/0, (b) 90/10, (c) 80/20, (d) 75/25; Figure S3. DSC traces of PPDO/PVPh blends of different compositions as marked (scanning rate = 20 °C/min); Figure S4. (a) Hydroxyl-stretching region for neat PVPh and PPDO/PVPh blend and (b) carbonyl stretching region for neat PPDO and PPDO/PVPh blend of different compositions.
