# Supplementary material for: Unique Periodic Rings Composed of Fractal-Growth Dendritic Branching in Poly(p-dioxanone)
Source: Polymers (Basel). 2022 Feb 19;14(4):805. doi: 10.3390/polym14040805 (PMC8963038; doi:10.3390/polym14040805)
Supplement: Supplementary file 1 [file polymers-14-00805-s001.zip › polymers-1566857-supplementary.pdf]

Supporting Information

Unique Periodic Rings Composed of Fractal-Growth Dendritic Branching in Poly(p-dioxanone)

Kuan-Ying Huang, Eamor M. Woo * and Selvaraj Nagarajan

Department of Chemical Engineering, National Cheng Kung University No. 1, University Road, Tainan 701-01, Taiwan; minnie850422@gmail.com (K.-Y.H.); nagarajan.tech@gmail.com (S.N.)
* Correspondence: emwoo@mail.ncku.edu.tw; Tel.: +886-6-275-7575 (ext. 62670)

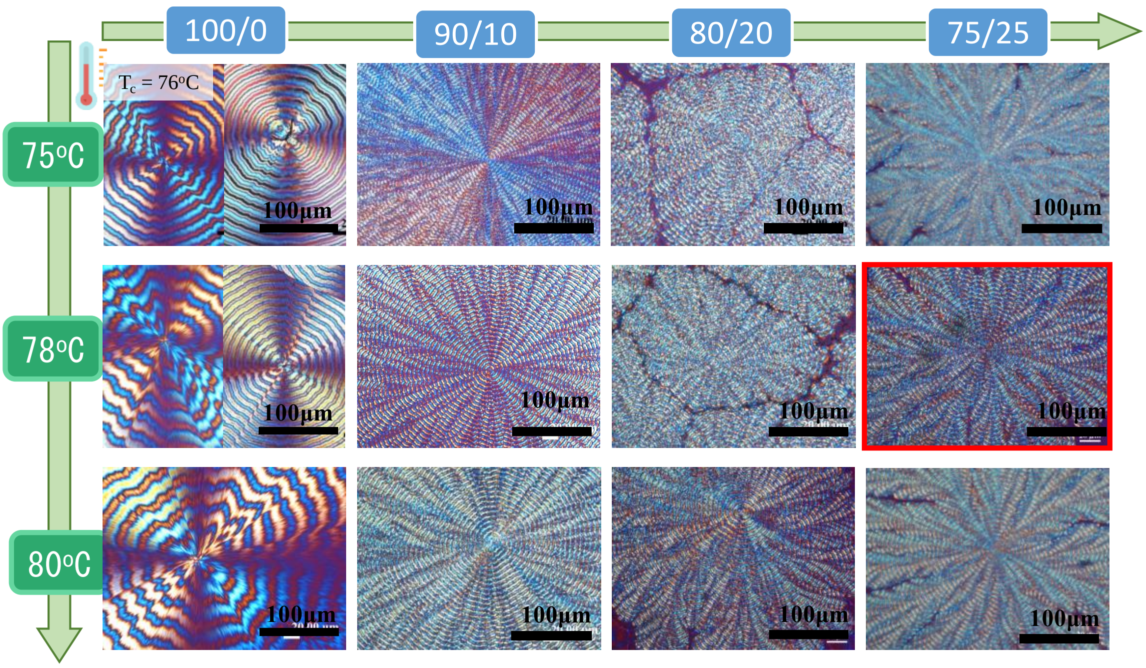

Figure S1. Effects of T_c and composition on the POM birefringence patterns of PPDO crystallized from PPDO/PVPh blend.

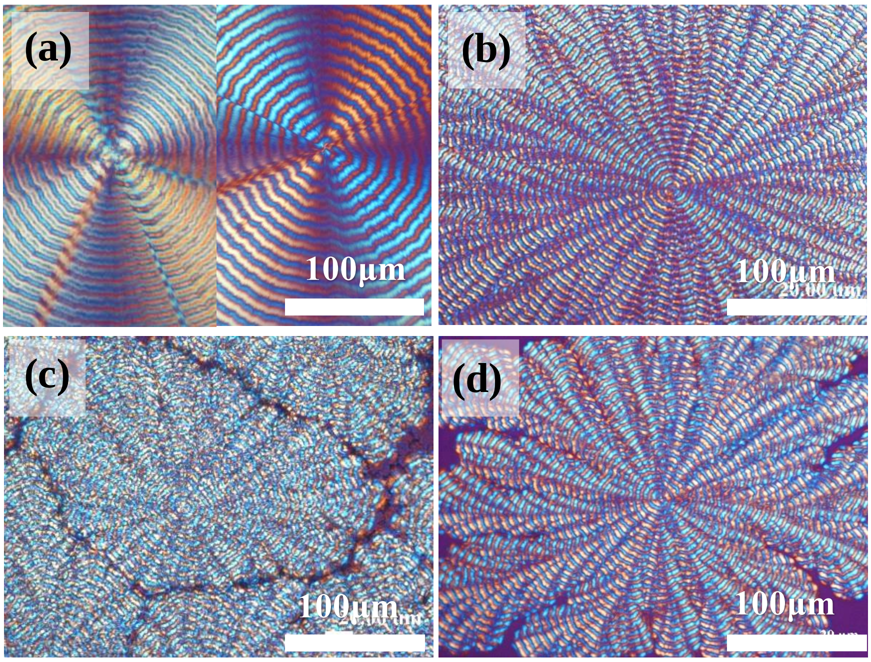

Figure S2. POM graphs of PPDO in PPDO/PVPh blend of four different compositions all crystallized at a specific T_c = 78 ^oC: (a) 100/0, (b) 90/10, (c) 80/20, (d) 75/25.

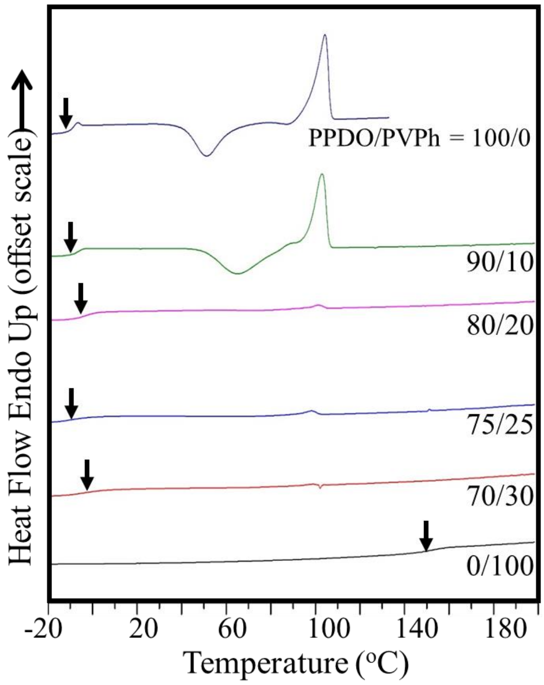

Figure S3. DSC trace of PPDO/PVPh blends of different compositions as marked (scanning rate = 20 °C/min). Note: these samples were quenched to amorphous states and immediately scanned. But PPDO/PVPh compositions (80/20, 75/ 25) can develop spherulitic crystals upon annealing at T_c=70-80°C after long period of times such as 3-4 days.

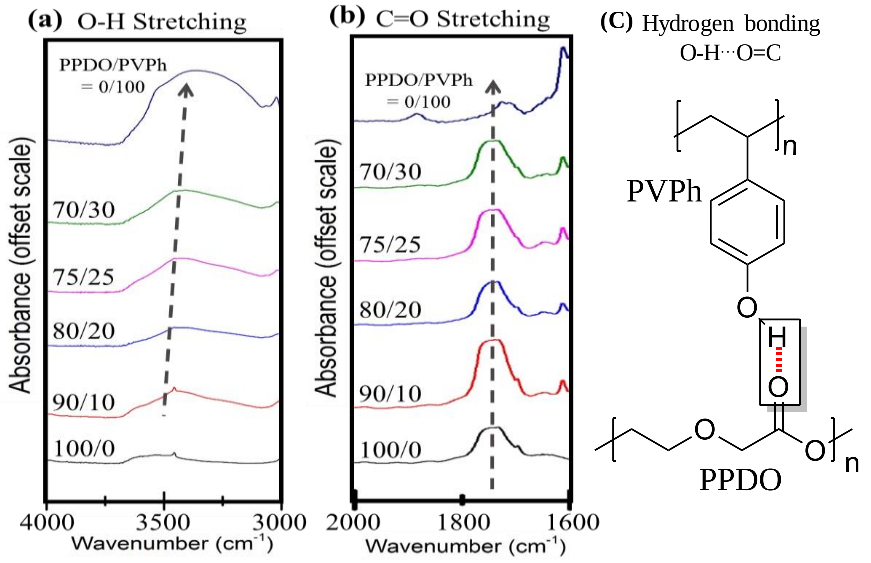

Figure S4. (a) Hydroxyl-stretching region for neat PVPh and PPDO/PVPh blend, (b) carbonyl-stretching region for neat PPDO and PPDO/PVPh blend of different compositions and (c) hydrogen bonding between PPDO and PVPh.
